# Highly Dispersed Cobalt Nanoparticles Embedded in Nitrogen-Doped Graphitized Carbon for Fast and Durable Potassium Storage

**DOI:** 10.1007/s40820-020-00534-x

**Published:** 2020-11-07

**Authors:** Xiaodong Shi, Zhenming Xu, Cheng Han, Runze Shi, Xianwen Wu, Bingan Lu, Jiang Zhou, Shuquan Liang

**Affiliations:** 1 School of Materials Science and Engineering, Central South University, Changsha, 410083 People’s Republic of China; 2 University of Michigan-Shanghai Jiao Tong University Joint Institute, Shanghai Jiao Tong University, Shanghai, 200240 People’s Republic of China; 3 Harvard John. A. Paulson School of Engineering and Applied Sciences, Harvard University, 29 Oxford Street, Cambridge, MA 02138 USA; 4 School of Chemistry and Chemical Engineering, Jishou University, Jishou, 416000 People’s Republic of China; 5 School of Physics and Electronics, State Key Laboratory of Advanced Design and Manufacturing for Vehicle Body, Hunan University, Changsha, 410082 People’s Republic of China; 6 Key Laboratory of Electronic Packaging and Advanced Functional Materials of Hunan Province, Central South University, Changsha, 410083 People’s Republic of China

**Keywords:** Cobalt nanoparticles, Nitrogen-doped graphitized carbon, Co–N bonds, Cycling stability, Potassium-ion batteries

## Abstract

Potassium-ion batteries (KIBs) have great potential for applications in large-scale energy storage devices. However, the larger radius of K^+^ leads to sluggish kinetics and inferior cycling performance, severely restricting its practical applicability. Herein, we propose a rational strategy involving a Prussian blue analogue-derived graphitized carbon anode with fast and durable potassium storage capability, which is constructed by encapsulating cobalt nanoparticles in nitrogen-doped graphitized carbon (Co-NC). Both experimental and theoretical results show that N-doping effectively promotes the uniform dispersion of cobalt nanoparticles in the carbon matrix through Co–N bonds. Moreover, the cobalt nanoparticles and strong Co–N bonds synergistically form a three-dimensional conductive network, increase the number of adsorption sites, and reduce the diffusion energy barrier, thereby facilitating the adsorption and the diffusion kinetics. These multiple effects lead to enhanced reversible capacities of 305 and 208.6 mAh g^−1^ after 100 and 300 cycles at 0.05 and 0.1 A g^−1^, respectively, demonstrating the applicability of the Co-NC anode for KIBs.

**Electronic supplementary material:**

The online version of this article (10.1007/s40820-020-00534-x) contains supplementary material, which is available to authorized users.

## Highlights

- Small cobalt nanoparticles are carefully encapsulated into a N-doped carbon shell (Co-NC) by calcining a Prussian blue analogue precursor.
- The presence of cobalt nanoparticles and Co-N bonds not only promotes adsorption behavior, but also reduces the diffusion energy barrier, enabling fast diffusion kinetics of K^+^ ions.
- The good diffusion kinetics and capacitive adsorption behavior of the Co-NC material synergistically contributes to enhanced potassium storage performances.

**Electronic supplementary material:**

The online version of this article (10.1007/s40820-020-00534-x) contains supplementary material, which is available to authorized users.

## Graphical abstract

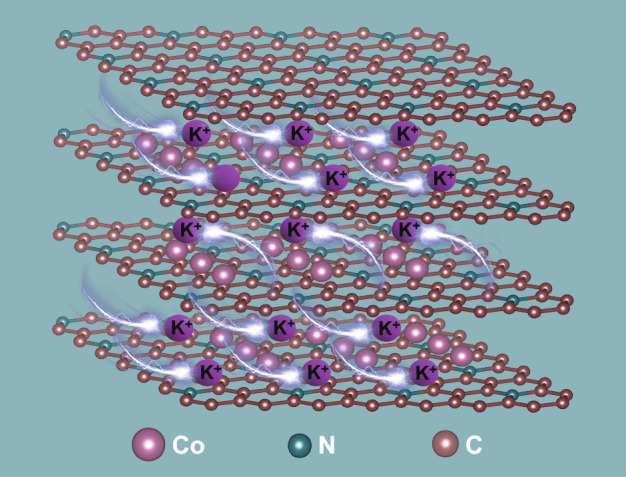

## Introduction

As emerging secondary energy storage devices, potassium-ion batteries (KIBs) have attracted extensive attention as alternatives to lithium-ion batteries (LIBs) because of the abundant potassium resources and the low redox potential of the K^+^/K couple [1–3]. Transition metal chalcogenides [4, 5], carbon materials [6, 7], phosphorus-based materials [8, 9], and alloy-based materials [10, 11] have been reported as anodes for KIBs. Among these systems, carbon materials such as graphitized and amorphous carbon have been considered as the most promising anodes because of their moldable structure and intrinsic electronic conductivity [12]. As a typical graphitized carbon material, graphite has been widely applied in LIBs. In KIBs, graphite anode presents a high capacity of 279 mAh g^−1^ and a distinct K^+^ (de)intercalation plateau above 0.1 V, suggesting its potential favorable applicability [13]. Unfortunately, graphitized carbon-based KIBs still suffer from large volume change during K^+^ intercalation [14], high potassiation energy barrier, and slow diffusion kinetics due to the larger radius of K^+^ [15], leading to a short cycle life and inferior rate capability.

Designing an adjustable structure is considered as an effective approach for reducing the volume change and thus enhancing the cycling stability. As reported, nanocage structures could relieve the volume strain of graphitized carbon during (de)intercalation of K^+^ [16], effectively contributing to a durable cycle performance. In addition, the nanospring structure of graphitized carbon also enables volume stress reduction and provides good cycling stability [17]. Moreover, employing a high-concentration electrolyte (HCE) is another valid strategy to improve the cycling stability of graphitized carbon. Potassium bis(fluorosulfonyl)imide (KFSI) in ethyl methyl carbonate (EMC, 1:2.5 molar ratio) has emerged as an efficient HCE for graphite anodes [18], where it generates a durable inorganic-rich solid electrolyte interphase (SEI) film and ensures superior cycling stability. Similarly, another kind of HCE (KFSI/1,2-dimethoxyethane (DME)/highly fluorinated ether in 1:1.9:0.95 molar ratio) can also ensure a long cycle life for KIBs because of the formation of a KF-rich SEI on the surface of the graphite anode [19]. Despite this progress, the harsh preparation conditions of the adjustable structure and the higher cost of the HCE still limit further applications of graphitized carbon anodes. Therefore, the development of new graphitized carbon materials for KIBs with high capacity, stable cycling behavior, and facile synthesis is a significant challenge.

Recently, porous graphitized carbon matrices decorated with transition metal species (Zn, Fe, Co, and Ni) have been derived from metal–organic frameworks or other three-dimensional (3D) precursors [20]. These materials have been widely applied in lithium-sulfur batteries [21, 22], lithium metal anodes [23], sodium metal anodes [24], zinc metal anodes [25], sodium-ion batteries [26], and oxygen reduction catalysts [27] because of their unique structure, high electrochemical activity, and abundant active sites. These multiple effects may improve the potassium storage capability of graphitized carbon anodes, which is an aspect worth further exploration.

Herein, cobalt nanoparticles wrapped by N-doped graphitized carbon (Co-NC) were synthesized by carbonizing the Prussian blue analogue (PBA) precursor of Zn_3_[Co(CN)_6_]_2_ and employed as anode for KIBs. As expected, Co-NC inherited the spherical morphology and porous structure of the PBA precursor, in which cobalt nanoparticles were uniformly dispersed and tightly encapsulated into N-doped graphitized carbon through the strong chemical interactions of the Co–N bonds. As a result, highly dispersed cobalt nanoparticles could cooperatively work with Co–N groups to regulate the electronic structure, enhance the electronic conductivity, and facilitate the charge transfer as well as the K^+^ adsorption. These multiple effects thus strengthen the diffusion kinetics and capacitive adsorption behavior of K^+^ ions, leading to a reversible capacity of 305 mAh g^−1^ at 0.05 A g^−1^ and a long-term cycling stability for up to 1000 cycles at 1 A g^−1^.

## Experimental Section

### Synthesis of Zn_3_[Co(CN)_6_]_2_ Precursor

Zn(CH_3_COO)_2_·2H_2_O (6 mmol), polyvinyl pyrrolidone (PVP, 7.2 g), and Pluronic F127 (4 g) were dissolved in 200 mL deionized water to form a transparent solution. Thereafter, K_3_[Co(CN)_6_]_2_ (4 mmol) was dissolved in another 200 mL of deionized water and added dropwise into the above solution under magnetic stirring and ultrasonic conditions in an ice-water bath. After aging the mixture for 24 h, the resulting white precipitate was collected and washed several times with deionized water. Finally, the Zn_3_[Co(CN)_6_]_2_ product was obtained by freeze-drying the white precipitate for another 12 h. All the chemicals are sourced from Shanghai Aladdin Bio-Chem Technology Co. LTD (China) and used directly without further refinement.

### Preparation of Co-NC and NC Samples

Co-NC composite was prepared by calcining the Zn_3_[Co(CN)_6_]_2_ precursor at 800 °C for 2 h under a H_2_/Ar atmosphere (5 vol% H_2_) at a heating rate of 4 °C min^−1^. As a reference, N-doped graphitized carbon (NC) was obtained by successively immersing the Co-NC composite into a 3 mol L^−1^ HF and 3 mol L^−1^ HCl solution under vigorous magnetic stirring for 12 h to remove most of the cobalt nanoparticles.

### Material Characterization

X-ray diffraction (XRD) patterns were obtained on a MiniFlex 600 instrument (Cu K_α_ radiation, *λ* = 0.154 nm, Rigaku Corporation, Japan). Scanning electron microscopy (SEM) (FEI Nova NanoSEM 230 m, FEI Corporation, United States of America), transmission electron microscopy (TEM) (Titan G2 60–300 with image corrector, FEI Corporation, United States of America), and high-resolution TEM (HRTEM) were used to determine the morphologies and crystal structures of the samples. X-ray energy-dispersive spectroscopy (EDS) was employed to obtain the elemental compositions. The degree of graphitization was evaluated by Raman spectroscopy (LabRAM HR800, Horiba Jobin Yvon Corporation, France), whereas Fourier transform infrared (FTIR) spectroscopy (Nicolet 6700, ThermoFisher Corporation, United States of America) was used to identify the coordination groups. Thermogravimetric (TG) analysis (Netzsch STA449C, Netzsch Corporation, Germany) was carried out to calculate the carbon content. Nitrogen adsorption–desorption isotherms and pore structures were analyzed using a multistation adsorption apparatus (Micromeritics ASAP 2460, Micromeritics Instrument Corporation, United States of America). X-ray photoelectron spectroscopy measurements were carried out on a ESCALAB 250Xi spectrometer (ThermoFisher Corporation, United States of America). X-ray absorption near-edge structure (XANES) spectra were obtained on the photoemission end-station at beamline BL10B of the National Synchrotron Radiation Laboratory (University of Science and Technology of China).

### Electrochemical Tests of KIBs

Co-NC, NC, and graphite anodes were prepared by grinding the active material (80%) with acetylene black (10%) and polyvinylidene fluoride binder (10%), followed by dispersing the resulting mixture in a specific amount of *N*-methyl pyrrolidone solution to form a black slurry. The slurry was then coated on Cu foil and dried in a vacuum oven at 80 °C for 12 h. Finally, CR2016-type coin cells were packed in a glove box with fresh potassium slices, 0.8 mol L^−1^ potassium hexafluorophosphate (KPF_6_) in ethylene carbonate:diethyl carbonate (EC:DEC), and glass fiber as the reference electrode, electrolyte, and separator, respectively.

Measurements of galvanostatic charge/discharge (GCD) profiles as well as cycle and rate performance were conducted on a LAND CT2001 test system (Wuhan LAND Electronic Co. Ltd, China) at the specific current densities. Cyclic voltammetry (CV) curves at different scan rates were obtained on a CHI660E electrochemical workstation (Shanghai Chenhua Instrument Co. LTD, China), and electrochemical impedance spectroscopy (EIS) measurements were carried out in the frequency range of 10 MHz–100 kHz. Finally, galvanostatic intermittent titration technique (GITT) data were collected on an Arbin BT2000 (Arbin Instrument Corporation, United States of America) to calculate the K^+^ diffusion coefficient (*D*_K_).

### Density Functional Theory (DFT) Calculations

All calculations were carried out using the projector-augmented wave method in the DFT framework, as implemented in the Quantum ESPRESSO software. The generalized gradient approximation and Perdew-Burke-Ernzerhof exchange functional were used in the calculations. The plane-wave energy cutoff was set to 30 Ry, and the Monkhorst–Pack method was employed for Brillouin zone sampling. The convergence criterion of the force calculations was set to 0.001 a.u. The NC model was built by replacing one carbon atom with a nitrogen atom in the 2 × 2 supercell of graphite, containing two carbon layers. The Co-NC model was prepared by stacking the Co (111) surface with the NC structure. The DFT-D2 method was used to account for the van der Waals interactions between the Co (111) surface and NC. To analyze the interactions between the K atoms and the NC structure, we calculated the formation energies of K atom insertion into the NC and Co-NC composites, using an insertion concentration K:C = 1:16. The formation energy (*E*_f_) was calculated as the energy difference of the system after and before the insertion process: *E*_f_ = *E*_*K inserted NC or Co-NC*_—*E*_*K*_—*E*_*NC or Co-NC*_, where *E*_*K inserted NC or Co-NC*_, *E*_*K*_, and *E*_*NC or Co-NC*_ represent the DFT energies of the K-inserted NC or Co-NC models, the energy of a K atom in the bulk, and the energy of the NC or Co-NC models, respectively. The energy barriers for K atom diffusion in NC or Co-NC were calculated using the nudged elastic band method.

## Results and Discussion

### Structural Characterizations

**Fig. 1:**
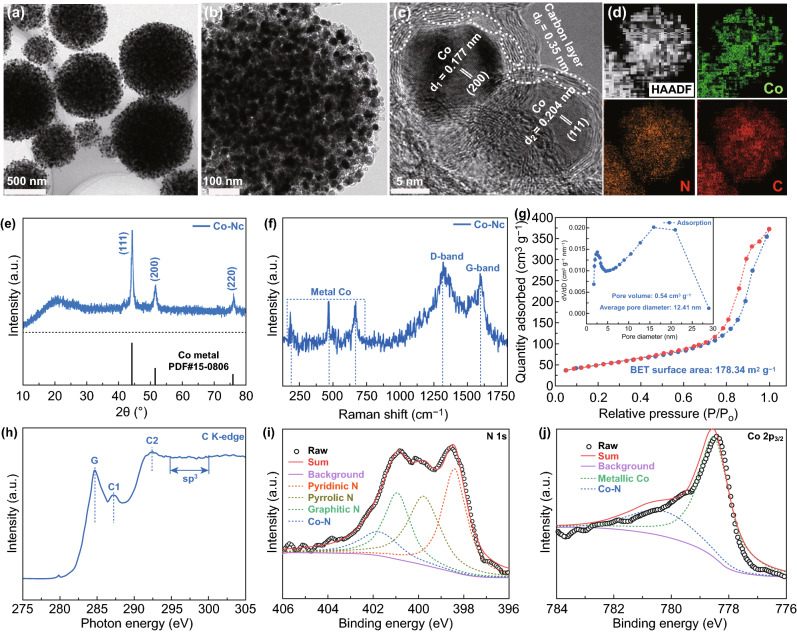
**a**, **b** TEM images, **c** HRTEM image, **d** mapping images, **e** XRD pattern, **f** Raman spectra, **g** N_2_ adsorption–desorption isotherm and pore distribution curves, **h** C K-edge XANES spectra, and **i**, **j** N 1 *s* and Co 2*p*_3/2_ spectra of Co-NC composite

The preparation process of the Co-NC sample is illustrated in Fig. S1. A spherical Zn_3_[Co(CN)_6_]_2_ precursor with a smooth surface, uniform size (500–800 nm, Fig. S2a), and porous structure (Fig. S2b, c) was synthesized through a facile co-precipitation reaction. In the subsequent calcination process, many CN^−^ groups transformed into N-doped carbon [28], while Co^2+^ ions were reduced to cobalt metal under H_2_/Ar atmosphere, simultaneously catalyzing the formation of a graphitized carbon layer [29]. A composite of cobalt nanoparticles and NC was then obtained (with a yield of approximately 44%, based on the TG curve in Fig. S2d). The SEM (Fig. S3a) and TEM (Fig. 1a, b) images show that high numbers of small nanoparticles composed of spherical products were cross-linked with a 3D porous network, inheriting the primary morphology and particle size of the PBA precursor. The HRTEM (Fig. 1c) and mapping (Fig. 1d) images of Co-NC further confirm that the individual nanoparticles were wrapped by a carbon layer with a certain degree of graphitization. The XRD pattern (Fig. 1e) shows three distinct diffraction peaks at 44.2°, 51.5°, and 75.9°, which could be indexed to the structure of cobalt metal (JCPDS card No. 15–0806). Moreover, the broad diffraction peak at 22.5° could be assigned to the (002) plane of carbon; the exact content of cobalt nanoparticles could be estimated as 33.8 wt% based on the TG curve in Fig. S3a, in agreement with the EDS results in Fig. S3b. Furthermore, Fig. 1f shows the Raman spectrum of the Co-NC sample. The two clear peaks at 1321.5 and 1595.3 cm^−1^ could be assigned to the D and G bands (with a moderate *I*_D_/*I*_G_ ratio value of 1.02), where *I*_D_ and *I*_G_ are the intensities of the D and G peaks, respectively. The three weaker peaks in the 200–700 cm^−1^ region were attributed to metallic cobalt. Fig. 1g illustrates the porous properties of Co-NC, showing a BET surface area of 178.34 m^2^ g^−1^ and an average pore size of 12.41 nm, which enables sufficient contact between electrode and electrolyte. In addition, as shown in Fig. S4, the BET surface area of the NC sample (222.3 m^2^ g^−1^) is slightly larger than that of the Co-NC sample, which can be ascribed to the formation of hollow carbon shells after the dissolution of cobalt nanoparticles in the concentrated acid solution.

The XANES technique, which is sensitive to electronic states and chemical bonds, was used to analyze the interfacial interactions in the Co-NC composite. As shown in Fig. 1h, the C K-edge spectra comprised three resonances located at 284.72 (G peak), 287.24 (C1 peak), and 292.42 eV (C2 peak). These peaks can be attributed to the dipole transition of the C 1* s* core electrons to π*_C = C,_ π*_C–N,_ and σ*_C–C_ antibonding states, respectively [30, 31]. These features confirm that the cobalt nanoparticles do not perturb the structure of the N-doped carbon shell, and additional C-N bonds form out of the graphene layer, introducing further *sp*^*3*^ interactions [32, 33]. The FTIR spectra in Fig. S3c also confirm the presence of C-N groups in the Co-NC composite [21, 27]. As shown in Fig. S3d, the Co L-edge XANES spectra of Co–NC show two peaks at 778.8 and 793.9 eV, corresponding to the L3 (2*p*_3/2_) and L2 (2*p*_1/2_) edges. The N-doping states were further investigated based on the survey spectrum in Fig. S3e, the high-resolution C 1* s* spectrum in Fig. S3f, and the high-resolution N 1* s* spectrum in Fig. 1i. The raw N 1* s* peak could be divided into four main components with binding energies of 398.4, 399.7, 400.9, and 401.8 eV, corresponding to pyridinic N, pyrrolic N, graphitic N, and Co–N bonds [27, 34]. On the other hand, the Co 2*p*_3/2_ peak could be deconvoluted into two components, corresponding to metallic Co (778.5 eV) and Co–N groups (780.5 eV) [27, 35]. The Co–N and C–N groups provide strong interfacial interactions between the core consisting of cobalt nanoparticles and the N-doped carbon shell, which promote the uniform dispersion of cobalt nanoparticles and enable adequate coating of the carbon layer [21, 36].

### Potassium Storage Performances

**Fig. 2:**
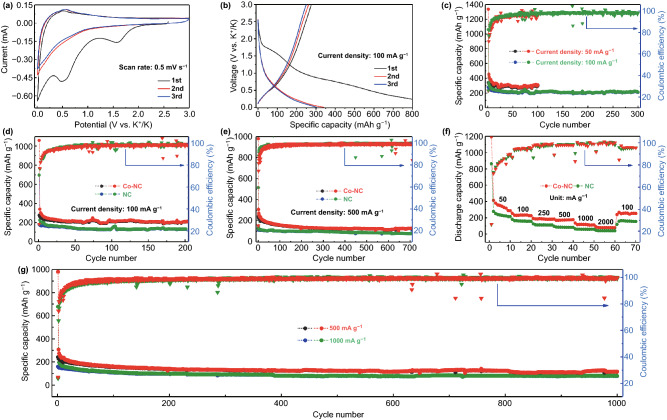
**a** CV curve of Co-NC anode at 0.5 mV s^−1^, **b** GCD curve of Co-NC anode at 100 mA g^−1^, **c** cycling performance and corresponding CE of Co-NC anode at 50 and 100 mA g^−1^, **d** cycling performance and corresponding CE of Co-NC and NC anodes at 100 mA g^−1^, **e** cycling performance and corresponding CE of Co-NC and NC anodes at 500 mA g^−1^, **f** rate performance and corresponding CE of Co-NC and NC anodes at 50, 100, 250, 500, 1000, 2000, and 100 mA g^−1^, **g** long-term cycling performance and corresponding CE of Co-NC anode at 500 and 1000 mA g^−1^

The potassium storage behavior of the Co-NC electrode was studied by CV and GCD measurements. Because cobalt is an electrochemically inert metal, it could not be alloyed with K^+^ ions. As shown in Fig. 2a, Co-NC exhibits the typical potassium storage behavior of the carbonaceous anode [12]. In particular, two distinct cathodic peaks appear at approximately 1.55 and 0.55 V in the initial intercalation of K^+^ ions and disappear in the subsequent scans, which could be attributed to the irreversible electrolyte decomposition and formation of the SEI layer [16, 37, 38]. On the other hand, the evident anodic peak at 0.52 V observed in the charge process could be ascribed to the deintercalation of K^+^ ions. Moreover, the good overlap between the CV curves of the second and third cycles suggests good cycling reversibility [39, 40]. The potential plateaus in the GCD curves shown in Fig. 2b are in good agreement with the peaks observed in the CV curves. In addition, Co-NC shows initial discharge and charge capacities of 1059.8 and 276.4 mAh g^−1^, respectively, indicating a low initial Coulombic efficiency (ICE) of 26.1%. The low ICE is due to the porous structure of Co-NC, which leads to a larger contact area between the electrode and electrolyte, resulting in a higher electrolyte consumption during the formation process of the SEI [41–44]. Fig. 2c illustrates the cycling performances of the Co-NC electrode at low current densities. The electrode delivers specific capacities of 305 and 208.6 mAh g^−1^ after 100 and 300 cycles at 0.05 and 0.1 A g^−1^, respectively. To investigate the favorable effects of the dispersed cobalt nanoparticles, we compared the cycle performances of Co-NC with those of NC and graphite anodes (Figs. S5, S6). As shown in Fig. 2d, e, Co-NC exhibits high reversible capacities of 208.4 and 129.4 mAh g^−1^ after 200 and 700 cycles at 0.2 and 0.5 A g^−1^, respectively; these values are higher than those of NC throughout the cycling process, indicating that the presence of cobalt nanoparticles could improve the potassium storage capability. Similar results were observed in the comparison of the rate capabilities (Fig. 2f), with Co-NC exhibiting average discharge capacities of 342.6, 264.2, 187.5, 175.4, 120.3, and 80.2 mAh g^−1^ at 0.05, 0.1, 0.25, 0.5, 1, and 2 A g^−1^, respectively. After restoring the current to 0.1 A g^−1^, Co-NC still delivers a capacity as high as 256.2 mAh g^−1^, denoting a good rate capability. Based on the long-term cycling performances (Fig. 2g) and GCD curves at various cycles (Fig. S7) of the Co-NC anode, the dispersed cobalt nanoparticles also provide low potential polarization and good cycling stability up to 1000 cycles, with reversible capacities of 115.7 and 78.5 mAh g^−1^ at 0.5 and 1 A g^−1^, respectively. Furthermore, as shown by the corresponding Coulombic efficiency (CE) results in Fig. 2c–g, all curves show a gradual upward trend in the initial stage and remain stable at approximately 99% after a certain number of cycles, denoting a good cycling reversibility. Furthermore, the effects of the calcination temperature and type of electrolyte on the potassium storage performance of the Co-NC anode were also investigated. As shown in Fig. S8, the specific capacity decreases with increasing calcination temperature, and the Co-NC anode obtained at 800 °C delivers a higher capacity and superior cycling stability. As shown in Fig. S9, the cells with ether-based electrolytes (1 mol L^−1^ KPF_6_ in DME and 1 mol L^−1^ KPF_6_ in diethylene glycol dimethyl ether) deliver a higher capacity as well as higher ICE values at the initial stage; however, the resulting cycling stability is unsatisfactory.

### Analysis of Potassium Storage Kinetics

**Fig. 3:**
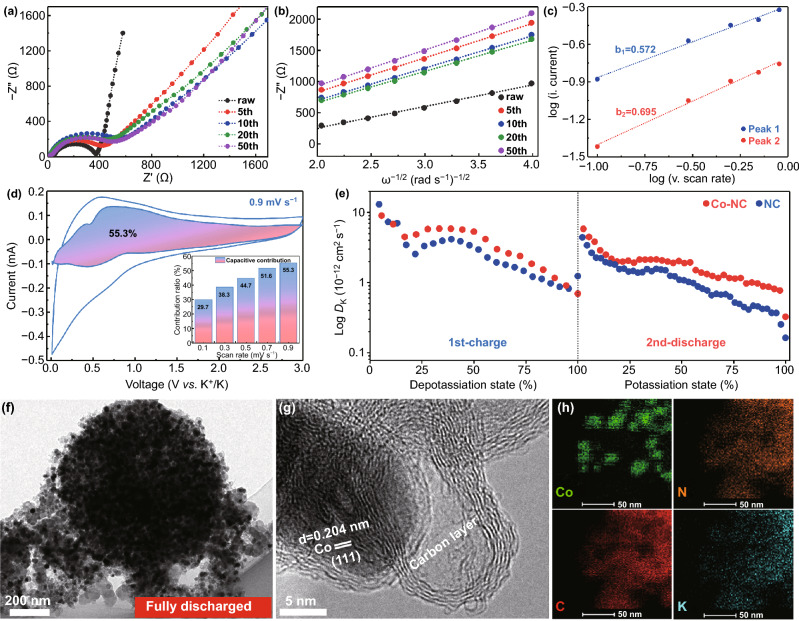
**a** Nyquist plots and **b** fitted linear *ω*^*−*1/2^ versus Z″ curves of Co-NC electrode after 0, 5, 10, 20, and 50 cycles; **c** corresponding log(*i*) *vs.* log(*v*) plots at specific peak currents; **d** capacitive contribution percentage at different scan rates and CV curve with capacitive fraction at 0.9 mV s^−1^ scan rate of Co-NC electrode; **e** GITT results of NC and Co-NC electrodes treated at 50 mA g^−1^; **f** TEM, **g** HRTEM, and **h** mapping images of Co-NC electrode at a fully discharged state

Based on the equivalent circuit models (Fig. S10) and analytical equations (Eqs. S1 and S2), the Nyquist plots and fitted *ω*^−1/2^*versus. -Z*″curves of the Co-NC and NC electrodes at different cycles in Figs. 3a, b and S11a, b were used to analyze differences in the electrochemical impedances and K^+^ diffusion coefficients. In short, Co-NC delivers a lower charge transfer resistance, reflecting an enhanced electronic conductivity, which improves the cycle performance at high rates. Moreover, we calculated the *D*_K_ values of the Co-NC and NC electrodes at different cycles (Eq. S3); these values, summarized in Table S1, highlight the faster diffusion kinetics of Co-NC, which is beneficial to the rate performance [45, 46]. Furthermore, the capacitive behavior of Co-NC is illustrated by the CV curves at different scan rates (Fig. S12); *b* values of 0.572 and 0.695 were calculated for the cathodic and anodic peaks of Co-NC, respectively (Fig. 3c, Eqs. S4 and S5); the two *b *values are close to 0.5, indicating that diffusion control is the main factor affecting K^+^ storage, whereas capacitance control is a minor factor [47, 48]. The capacitive contribution ratio of Co-NC could be quantitatively determined as shown in Fig. 3d Eqs.6 and 7), which show that this contribution increases from 29.7 to 55.3% as the scan rate increases 0.1 to 0.9 mV s^−1^. The moderate capacitive behavior could be attributed to the Co–N groups and nitrogen dopants at the interface of cobalt nanoparticles and graphitized carbon, which introduce additional adsorption sites and accelerate the reaction kinetics of Co-NC [49–51].

The diffusion kinetics of graphite, Co-NC, and NC electrodes were investigated by GITT (Figs. S13, S14, and Eq. S8). As shown in Fig. 3e, the Co-NC electrode delivers a higher K^+^ diffusion coefficient than NC and graphite throughout the (de)potassiation process, which is in good agreement with the EIS results. These results further demonstrate that the presence of cobalt nanoparticles, nitrogen dopants, and Co–N bonds effectively enhances the electronic conductivity, generates active sites, and promotes the charge transfer behavior, synergistically endowing the Co-NC anode with a faster diffusion kinetics [52, 53]. Moreover, the morphology and composition of the Co-NC electrode at fully discharged and charged states were characterized by TEM, HRTEM, and EDS, to confirm its structural evolution. As shown in Figs. 3f, g and S15a, b, the stable core–shell structure of Co-NC allows its spherical morphology to be maintained without apparent structural collapse during the (de)intercalation process, contributing to the long-term cycling ability. Moreover, by combining the mapping (Fig. 3h) and EDS (Fig. S16a) results with the ex situ XRD patterns (Fig. S16b), we further confirmed that the core of the cobalt nanoparticles remains stable and is not alloyed with K^+^ during the charge and discharge processes. Therefore, the potassium storage capacity of the Co-NC anode may originate from the intercalation of K^+^ ions in the graphitized carbon layer and the adsorption of K^+^ ions at the N-doping active sites, as well as at the interfaces between cobalt nanoparticles and graphitized carbon connected by Co–N groups.

**Fig. 4:**
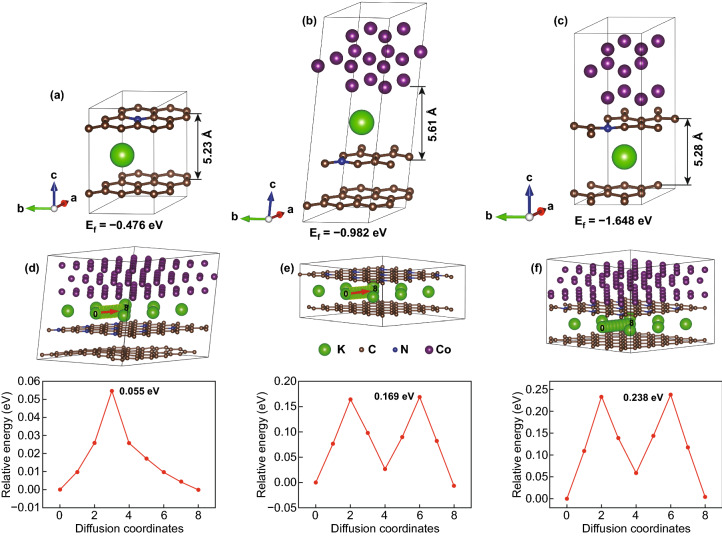
Adsorption energy of K^+^ ion **a** at the carbon layer of NC, **b** at the interface of cobalt nanoparticles and NC, and **c** at the carbon layer of Co-NC; corresponding barrier energy for K^+^ ions diffusing **d** at the interface of cobalt nanoparticles and NC, **e** between the carbon layers of NC, and **f** between the carbon layers of Co-NC

DFT calculations were performed to further elucidate the adsorption and diffusion properties of K^+^ ions in the NC and Co-NC electrodes. As displayed in Fig. S17, Co-NC shows a higher density of states than the NC model around the Fermi level, confirming its enhanced electronic conductivity [54]. As shown in Fig. 4a–c, the adsorption energies of K^+^ at the carbon layer of NC, at the cobalt nanoparticle/NC interface, and at the carbon layer of Co-NC, were calculated as −0.476, −0.982, and −1.648 eV, respectively. In short, the *E*_f_ value of Co-NC is higher than that of NC, while the *E*_f_ value of Co-NC at the interface is lower than that at the carbon layer, indicating the enhanced adsorption of K^+^ ions by Co-NC and the preference of these ions for the adsorption sites at the carbon layer [14, 55]. The diffusion energy barriers of K^+^ ions in the NC and Co-NC models are displayed in Fig. 4d–f, which show K^+^ ions diffusing at the interface of cobalt nanoparticles and N-doped graphitized carbon presents the lowest energy barrier, reflecting an enhanced diffusion kinetics in Co-NC [56]. These results indicate that the presence of cobalt nanoparticles could not only promote the adsorption and increase the number of adsorption sites, but also reduce the diffusion energy barrier and facilitate the diffusion kinetics.

## Conclusion

In summary, a Co-NC hybrid was prepared by calcining a Zn_3_[Co(CN)_6_]_2_ precursor and used as anode for KIBs. During the calcination process, abundant CN^−^ groups transform into N-doped carbon, while massive Co^2+^ ions are converted into small cobalt metal particles, simultaneously catalyzing the formation of graphitized carbon and core–shell structures. As a result, the presence of Co–N bonds ensures the tight encapsulation of cobalt nanoparticles into NC. Moreover, the highly dispersed cobalt nanoparticles could synergistically interact with Co–N bonds to develop a 3D conductive network, enhance the electronic conductivity, and provide effective ion diffusion and charge transfer pathways. These multiple advantages effectively facilitate the adsorption and (de)intercalation kinetics of K^+^ ions, further delivering an enhanced reversible capacity of 305 mAh g^−1^ at 0.05 A g^−1^ and a good rate capability of 80.2 mAh g^−1^ at 2 A g^−1^. This study may provide new insights into the structural design of graphitized carbon materials with fast diffusion kinetics and durable cyclic performance in batteries based on potassium and other alkali metal ions.

## Electronic supplementary material

Below is the link to the electronic supplementary material.

Supplementary file1 (PDF 1802 kb)
