# Supplementary material for: Highly Dispersed Cobalt Nanoparticles Embedded in Nitrogen-Doped Graphitized Carbon for Fast and Durable Potassium Storage
Source: Nanomicro Lett. 2020 Nov 7;13:21. doi: 10.1007/s40820-020-00534-x (PMC8187504; doi:10.1007/s40820-020-00534-x)
Supplement: Supplementary file 1 — Supplementary file1 (PDF 1802 kb) [file 40820_2020_534_MOESM1_ESM.pdf]

Supporting Information for

Highly Dispersed Cobalt Nanoparticles Embedded in Nitrogen-Doped Graphitized Carbon for Fast and Durable Potassium Storage

Xiaodong Shi [1], Zhenming Xu [2, 3], Cheng Han [1], Runze Shi [1], Xianwen Wu [4, *], Bingan Lu [5], Jiang Zhou [1, 6, *], Shuquan Liang [1, 6]

[1]School of Materials Science and Engineering, Central South University, Changsha 410083, People's Republic of China

[2]University of Michigan-Shanghai Jiao Tong University Joint Institute, Shanghai Jiao Tong University, Shanghai 200240, People's Republic of

[3]Harvard John. A. Paulson School of Engineering and Applied Sciences, Harvard University, 29 Oxford Street, Cambridge, Massachusetts, 02138, USA

[4]School of Chemistry and Chemical Engineering, Jishou University, Jishou 416000, People's Republic of China.

[5]School of Physics and Electronics, State Key Laboratory of Advanced Design and Manufacturing for Vehicle Body, Hunan University, Changsha 410082, People's Republic of China

[6]Key Laboratory of Electronic Packaging and Advanced Functional Materials of Hunan Province, Central South University, Changsha 410083, People's Republic of China

*Corresponding authors. E-mail: wxwcsu2011@163.com (Xianwen Wu); zhou_jiang@csu.edu.cn (Jiang Zhou)

S1 Relevant Calculation Formulas

S1.1 Calculating the K^+ Diffusion Coefficient Based on EIS Data

Nyquist plot of EIS is composed by two parts: (a) a straight line at low frequency region is ascribed to Warburg impedance (W_s), which has close relation with the diffusion coefficient of K^+ ions; (b) a semicircle at middle-high frequency region is corresponding to the resistances of active material, mainly including the contact resistance (R_s) and charge transfer resistance (R_{ct}).

$$\omega=2\pi f \quad \text{(S1)}$$

$$Z''=R+\sigma\omega^{-1/2} \quad \text{(S2)}$$

$$D = 0.5R^2T^2/S^2n^4F^4C^2\sigma^2 \quad \text{(S3)}$$

According to Eqs. S1 and S2, the corresponding slopes (σ) could be determined through fitting of Z'' with $\omega^{-1/2}$. For Formula S3, R is the gas constant (8.314 J mol^{-1} K^{-1}), T is Kelvin temperature (293.15 K), S is the area of working electrode (1.13 cm^2), n is the electronic

transfer number, F is the Faraday constant (96485 C mol^{-1}) and C is the concentration of K^+ ions in the lattice (roughly estimated as electrolyte concentration).

S1.2 Calculating the *b* Value and Capacitive Contribution Ratio

The response current in the CV plot obeys the relationship of Equation S4. As described in Formula S5, the parameter of b could be calculated as the slope value of the fitting linear of *log(i) vs. log(v)*.

$$i = a\, v^b \qquad \text{(S4)}$$

$$\log (i) = \log a + b \log (v) \qquad \text{(S5)}$$

According to the Formula S6, the response current (i) could be divided into two components by introducing new parameters of k_1 and k_2.

$$i = k_1 v + k_2 v^{1/2} \qquad \text{(S6)}$$

$k_1 v$ originates from the capacitive contribution, $k_2 v^{1/2}$ originates from the diffusion-limited Faradaic processes. Based on Formula S7, k_1 value could be acquired through the linear relationship between $i/v^{1/2}$ and $v^{1/2}$.

$$i/v^{1/2} = k_1 v^{1/2} + k_2 \qquad \text{(S7)}$$

S1.3 Calculating the K$^+$ Diffusion Coefficient Based on GITT Data

The diffusion coefficient on basis of GITT data is calculated by the following formula:

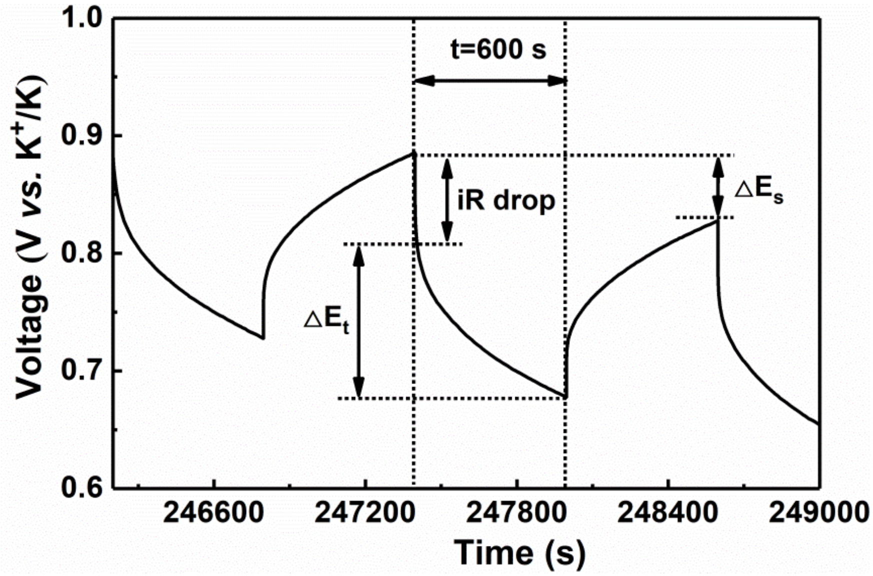

$$D = \frac{4L^2}{\pi t}\left(\frac{\Delta E_s}{\Delta E_t}\right)^2 \qquad \text{(S8)}$$

t is the duration of the current pulse (600 s). L is potassium ion diffusion length (cm). For compact electrode, it is equal to thickness of electrode. ΔEs is the quasi-thermodynamic equilibrium potential difference before and after the current pulse, ΔE_t is the potential difference during current pulse.

S2 Supplementary Figures and Tables

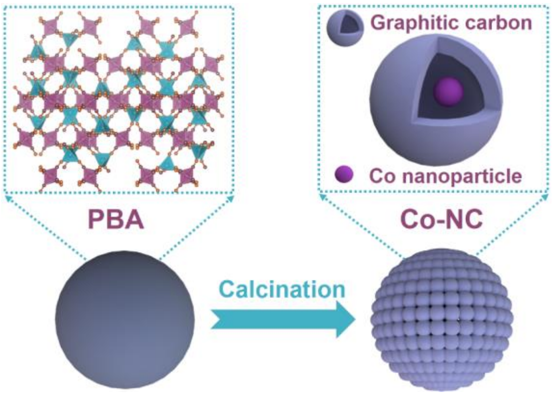

Fig. S1 Schematic illustration the preparation process of Co-NC composite

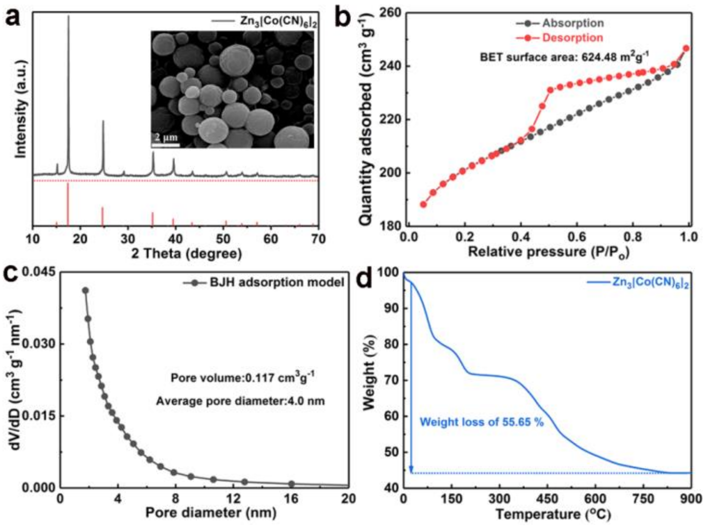

Fig. S2 (**a**) SEM image and XRD pattern, (**b**) Isothermal N_2 adsorption-desorption profile, (**c**) Pore size distribution curve and (**d**) TG curve of $Zn_3[Co(CN)_6]_2$ precursor

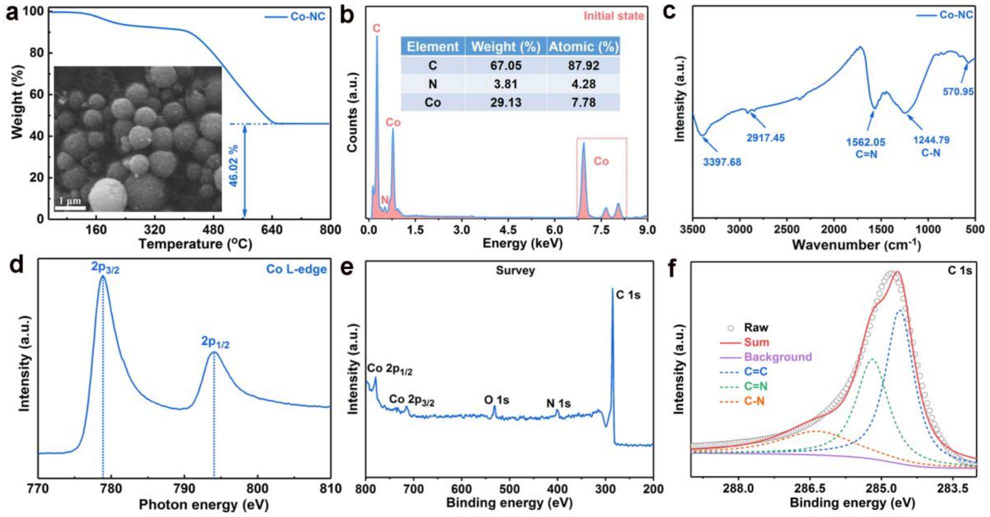

Fig. S3 (**a**) SEM image and TG curve, (**b**) EDS element content, (**c**) FTIR spectra, (**d**) Co L-edge XANES spectra, (**e**) Survey spectra and (**f**) C 1s spectra of Co-NC composite

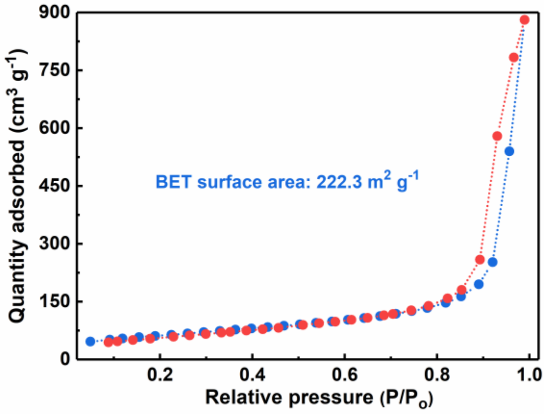

Fig. S4 N_2 adsorption-desorption isothermal curve of NC sample

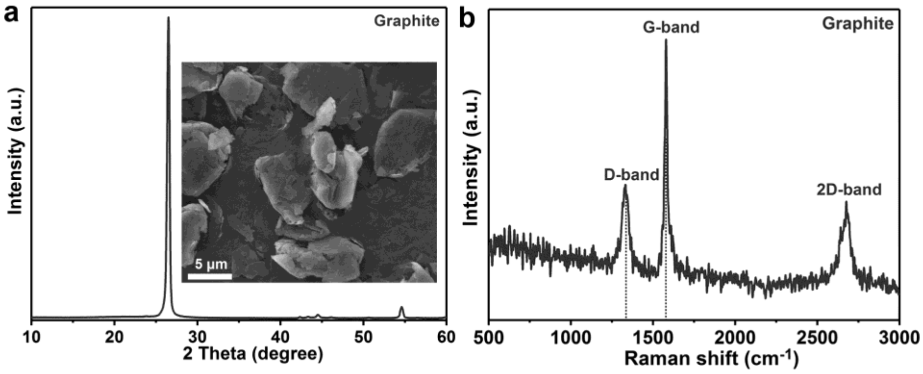

Fig. S5 (**a**) SEM image and XRD pattern, (**b**) Raman spectra of graphite

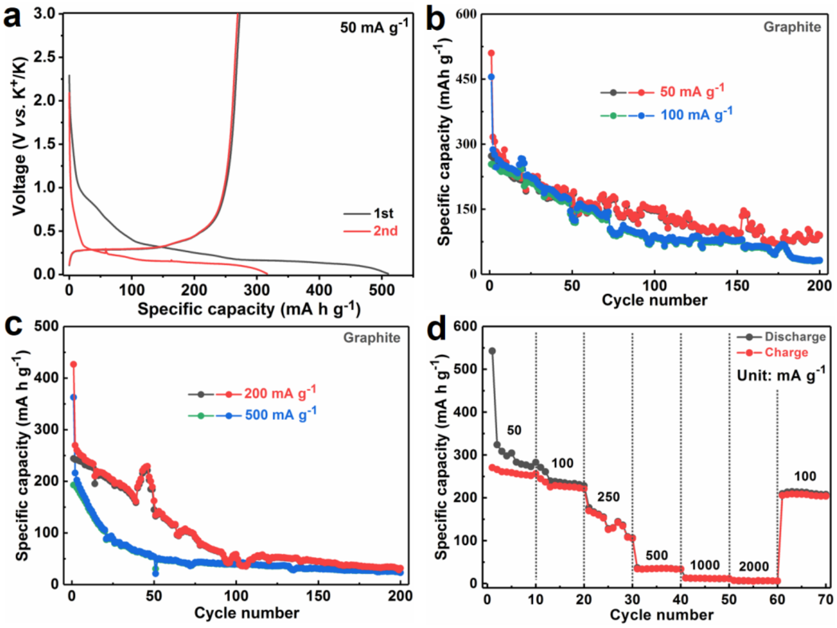

Fig. S6 (**a**) GCD curve of graphite anode at 50 mA g^{-1}; (**b**) Cycling performance of graphite anode at 50 and 100 mA g^{-1}; (**c**) Cycling performance of graphite anode at 200 and 500 mA g^{-1}; (**d**) Rate capability of graphite anode at different current densities

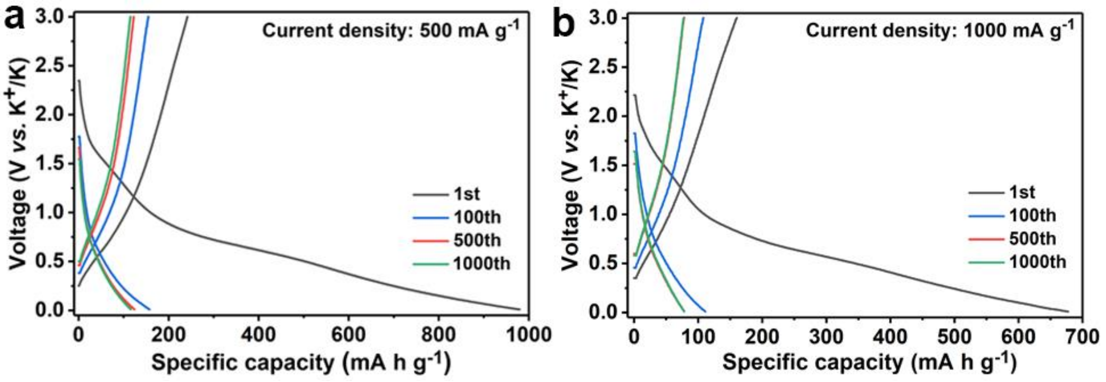

Fig. S7 GCD curves of Co-NC anode at various cycles (1st, 100th, 500th, and 1000th) under the current density of (**a**) 500 mA g^{-1} and (**b**) 1000 mA g^{-1}

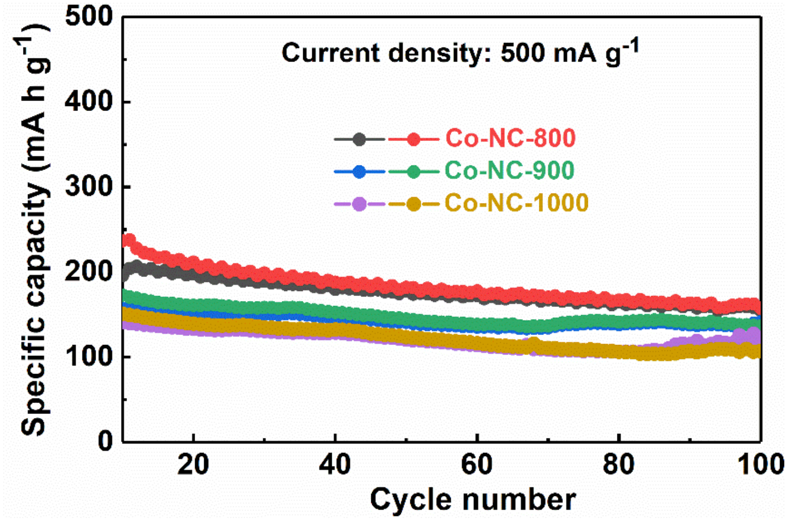

Fig. S8 Cycling performances of different Co-NC anodes at 500 mA g^{-1} from 10 to 100 cycles

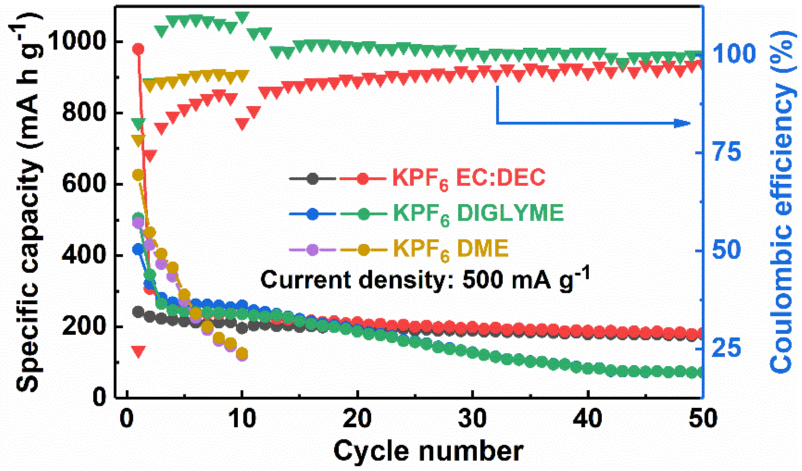

Fig. S9 Cycling performances of Co-NC anode with different KPF_6-based electrolytes at 500 mA g^{-1}

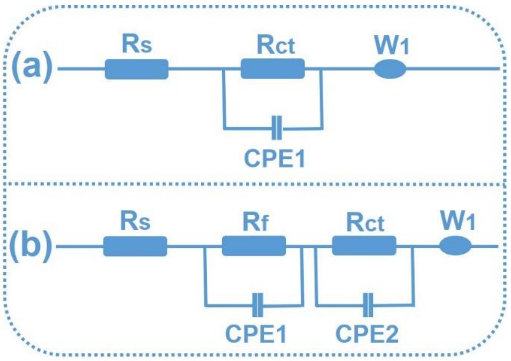

Fig. S10 Equivalent circuit models for the EIS results tested at (**a**) initial state and (**b**) after certain cycles

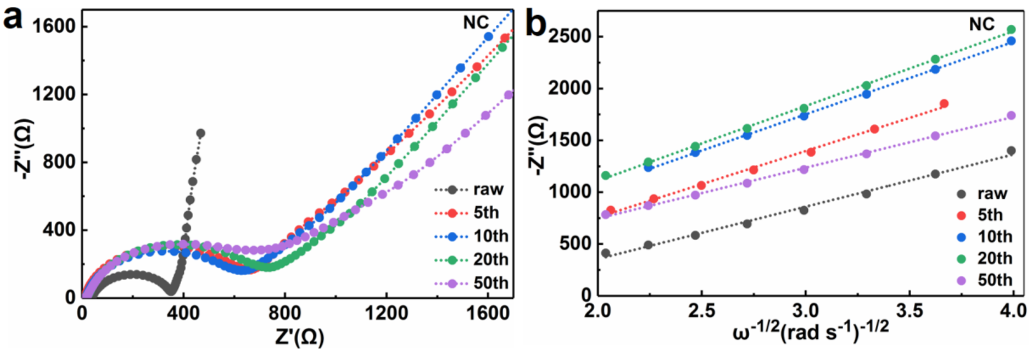

Fig. S11 (**a**) Nyquist plots and (**b**) the fitted curves of $\omega^{-1/2}$ *vs.* $-Z''$ of NC electrode after 0, 5, 10, 20, and 50 cycles

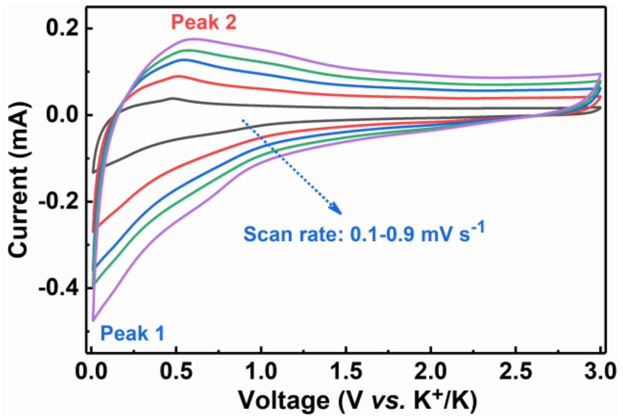

Fig. S12 CV curves of Co-NC electrode at different scan rates from 0.1 to 0.9 mV s^{-1}

Table S1 Diffusion coefficients of Co-NC and NC electrode at different cycles

Diffusion coefficients	Cycle numbers	Co-NC	NC
	0	3.51	1.64
	5	1.38	1.02
D_K [10^{-13} cm^2 s^{-1}]	10	1.57	0.857
	20	1.67	0.804
	50	1.86	1.74

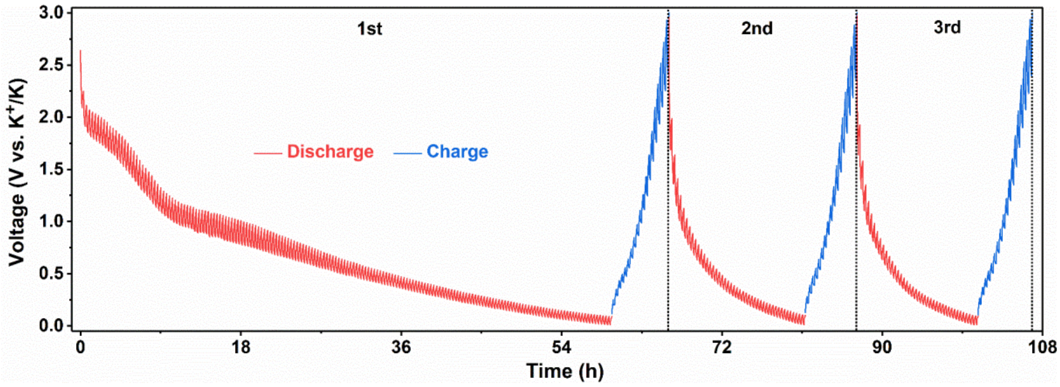

Fig. S13 Voltage-time profiles of Co-NC electrode tested by GITT at 50 mA g^{-1}

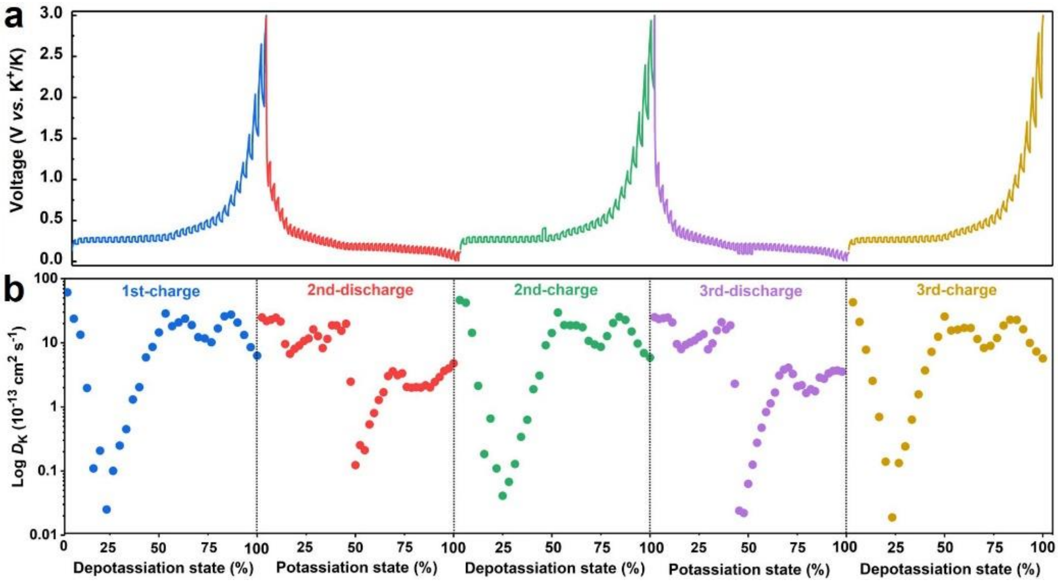

Fig. S14 (**a**) Voltage-time profile tested at 50 mA g^{-1} and (**b**) K^+ diffusion coefficient (D_k) of graphite anode calculated by GITT

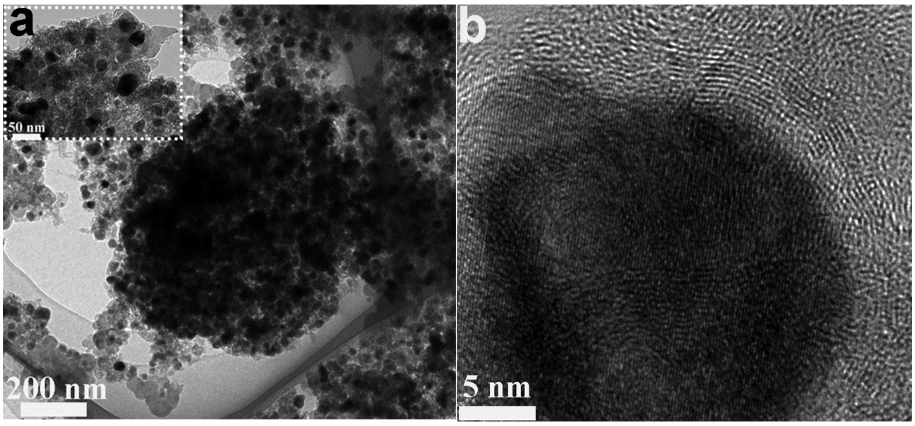

Fig. S15 (**a**) TEM image and (**b**) HRTEM image of Co-NC electrode at fully charged state

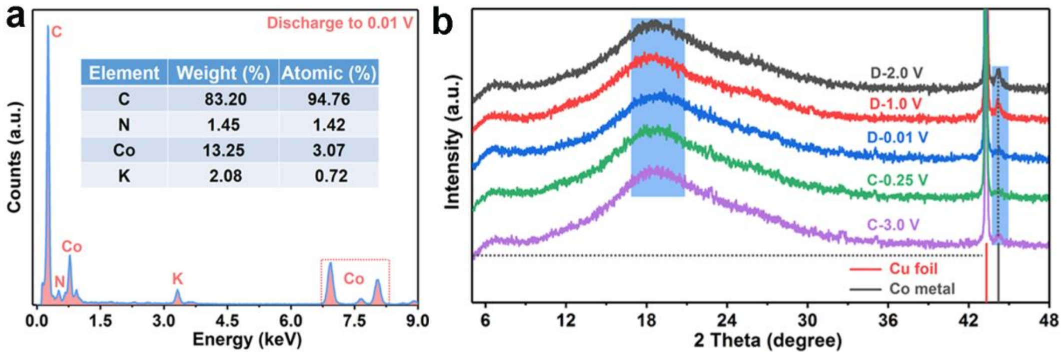

Element	Weight (%)	Atomic (%)
C	83.20	94.76
N	1.45	1.42
Co	13.25	3.07
K	2.08	0.72

Fig. S16 (**a**) EDS element content of Co-NC electrode at fully discharged state; (**b**) *ex situ* XRD of Co-NC electrode

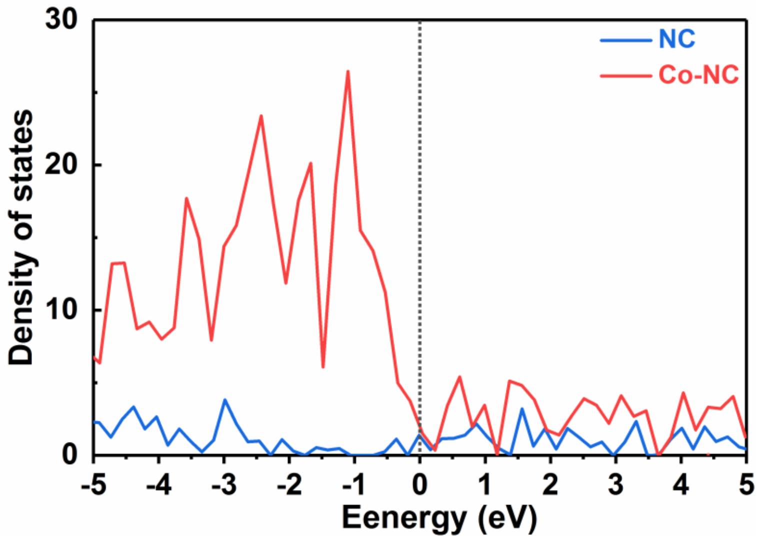

Fig. S17 Density of state (DOS) of NC and Co-NC models
